# Epiphyte type and sampling height impact mesofauna communities in Douglas-fir trees

**DOI:** 10.7717/peerj.5699

**Published:** 2018-10-12

**Authors:** Alexander R. Young, Jesse E.D. Miller, John Villella, Greg Carey, William R. Miller

**Affiliations:** 1 Department of Forest and Natural Resource Management College of Environmental Science and Forestry, State University of New York (SUNY), Syracuse, NY, United States of America; 2 Department of Environmental Science and Policy, University of California, Davis, CA, United States of America; 3 Siskiyou Biosurvey, Ashland, OR, United States of America; 4 Department of Biology and Chemistry, Baker University, Baldwin City, KS, United States of America

**Keywords:** Tardigrade, Canopy, Epiphyte, Microfauna, Microclimate

## Abstract

Branches and boles of trees in wet forests are often carpeted with lichens and bryophytes capable of providing periodically saturated habitat suitable for microfauna, animals that include tardigrades, rotifers, nematodes, mites, and springtails. Although resident microfauna likely exhibit habitat preferences structured by fine-scale environmental factors, previous studies rarely report associations between microfaunal communities and habitat type (e.g., communities that develop in lichens vs. bryophytes). Microfaunal communities were examined across three types of epiphyte and three sampling heights to capture gradients of microenvironment. Tardigrades, rotifers, and nematodes were significantly more abundant in bryophytes than fruticose lichen or foliose lichen. Eight tardigrade species and four tardigrade taxa were found, representing two classes, three orders, six families, and eight genera. Tardigrade community composition was significantly different between bryophytes, foliose lichen, fruticose lichen, and sampling heights. We show that microenvironmental factors including epiphyte type and sampling height shape microfaunal communities and may mirror the environmental preferences of their epiphyte hosts.

## Introduction

Tree canopies house a tremendous diversity of life and create horizontal and vertical heterogeneity in forest ecosystems (Nadkarni, 1994; Lowman & Rinkner, 2004). Major forms of life in temperate canopies include mammals, birds, and epiphyte mats. In addition to large organisms, small organisms occupy canopy soil and epiphyte material including the phyla Tardigrada, Rotifera, and Nematoda (collectively microfauna), which are often overlooked in forest ecology and biodiversity studies (Glime, 2013; Voegtlin, 1982; Wilson, 2002). Microfauna living in bryophytes and lichens are affected by humidity and condensation and will desiccate without regular precipitation and re-animate when sufficient moisture returns (Kinchin, 1994; Nelson, 1982; Boothby et al., 2017). The global distribution of tardigrades, rotifers, and nematodes in ecosystems, and the roles they play as primary and secondary consumers of canopy food webs, make them useful for comparing micro-scale community dynamics (Sánchez-Moreno, Ferris & Guil, 2008; Collins & Bateman, 2001; Harada & Ito, 2006; Sohlenius, Boström & Jönsson, 2004). However, the extent to which geographic variation in microfaunal communities is due to random dispersal processes, or is a reflection of habitat suitability, remains an open question. Relatively few ecological studies explore the impact of macro and micro environmental factors impact on microfaunal communities (Guil et al., 2009; Mitchell, Miller & Davis, 2009; Chang et al., 2015; Porazińska et al., 2012; Zawierucha et al., 2015).

Forest canopies create vertical gradients of microclimate which can be characterized by heterogeneity in humidity, sunlight, airflow, and nutrient availability (Geiger, 1967). These forces result in stratified habitats with distinct microclimates and create an elegant system for examining microclimate factors associated with canopy height, and differences between epiphyte habitat (McCune, 1993; Donoso, Johnston & Kaspari, 2010). Gradients of light availability, CO_2_ concentration, humidity, and desiccation rates occur along vertical axes in tree canopies with tree tops experiencing higher light availability and lower humidity (McCune, 1993; Geiger, 1967). The “similar gradients hypothesis” suggests that drivers of epiphyte distribution such as forest age, regional precipitation, and the vertical gradient of height could also impact other canopy organisms by creating similar habitat attributes or dispersal methods via different causal mechanisms (McCune et al., 1997). Furthermore, while previous studies suggest macro-environmental factors (e.g., elevation) shape macrofaunal communities, this response may be elicited indirectly through changes in vegetation communities or changes in habitat growth form which elicit microfaunal community response, rather than altitude directly (Wright, 1991; Jönsson, 2003; Richardson, Richardson & Soto-Adames, 2005; Guil et al., 2009; Zawierucha et al., 2016).

Studies report contrasting microfaunal responses to macro-environmental factors (Dastych, 1987; Nelson, 1975; Kathman & Cross, 1991; Young & Clifton, 2015). The factors governing small-scale distributions of microfauna remain elusive despite our awareness of their global distribution (McInnes, 1994). Local micro-environmental factors such as humidity may be more informative than regional factors such as elevation to explain species distribution due to taxa-level micro-climate suitability (Collins & Bateman, 2001; Guil et al., 2009; Degma, Katina & Sabatovicova, 2011; Zawierucha et al., 2015). For example, studies conducted on mountain slopes show both positive and negative impacts of altitude on tardigrade communities (Dastych, 1987; Beasley, 1988; Collins & Bateman, 2001; Zawierucha et al., 2015) while laboratory based studies support groupings of tardigrade species based on varying affinities for moisture (Ramazzotti & Maucci, 1983; Wright, 1989). Additionally, in one of the few studies of tardigrade phenology, Schuster & Greven (2013) tracked the body length and reproduction statistics including # of gravid females over 5 years and found that humidity was negatively correlated with # of oocytes and juveniles, while # of hours of sunlight was negatively correlated with body length and the percentage of juveniles present (Schuster & Greven, 2013). Field studies have an important place in advancing research on tardigrada, specifically in directing efforts to better culture tardigrade taxa in laboratory settings.

Tardigrades, nematodes and rotifers exhibit differences in stress tolerant dormant stages associated with dispersal strategies which support varying habitat suitability for taxa that can persist after arrival (Bongers & Ferris, 1999; Fontaneto, Melone & Ricci, 2003; Guil, Sánchez-Moreno & Machordom, 2008; Ramazzotti & Maucci, 1983; Wright, 1989). Nematodes vary in their sensitivity to disturbance but have been shown to have higher abundance with increasing soil porosity (Harada & Ito, 2006; Bongers & Ferris, 1999) and increased mortality rates with higher soil salinity (Poage et al., 2008). Rotifers display geographic distributions that suggest habitat preference, although species distributions were highly variable (Fontaneto & Ricci, 2006). Tardigrades were linked to tree species substrate and demonstrated higher abundance in the tops of trees in a mixed deciduous forest in Kansas, U.S.A. (Mitchell, Miller & Davis, 2009; Miller, Gallardo & Clark, 2013; Chappell et al., 2015; Chang et al., 2015). Also, tardigrade communities in leaf litter of Beech forests in Modenese Apennine (Italy) and Roan Mountain (TN, U.S.A.) had similar species composition (Guidetti, Bertolani & Nelson, 1999). Challenges to understanding limno-terrestrial microfaunal ecology include their patchy distributions (Meyer, 2006) and the difficulty of species identification which may be influenced by ontogeny, cryptic species diversity, and a poor understanding of population clustering (Miller, Miller & Heatwole, 1994; Degma, Katina & Sabatovicova, 2011; Morek et al., 2015).

**Figure 1:**
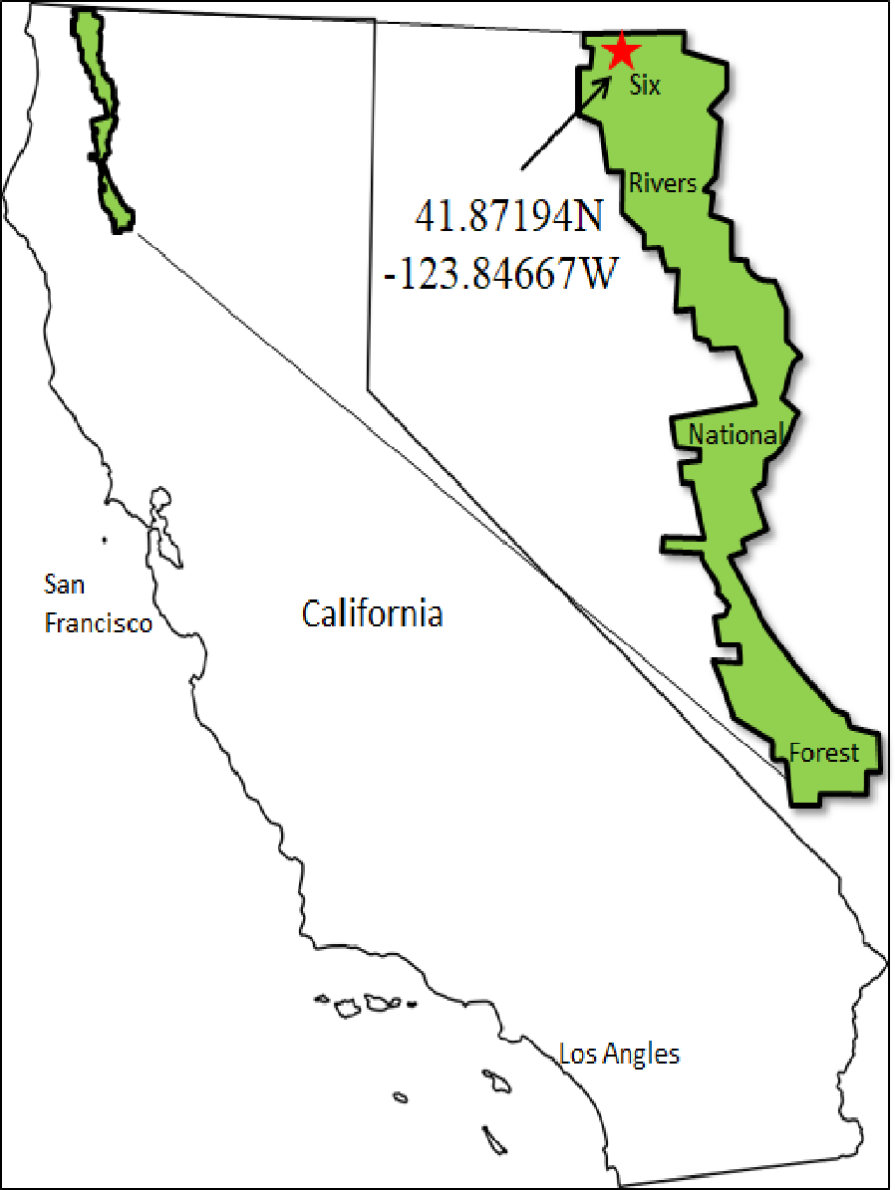
A map of the study area.

In this study, we document the density and diversity of tardigrades, rotifers, and nematodes in a Douglas-fir forest canopy in Northern California, USA (Fig. 1). To test the factors of epiphyte type and abiotic factors associated with height, we collected a factorial combination of multiple epiphyte types at multiple sampling heights in nine Douglas-fir trees. We expect microfaunal populations to respond to epiphyte type and sampling height because of the differences in water availability which represent barriers to micro-population establishment.

## Methods

### Site description

Field sampling took place in September, 2015 in Six Rivers National Forest of California, USA (41.871, −123.846). The site has a Mediterranean climate with an annual average annual precipitation of 200 cm. The soil is primarily composed of deep, well-drained soils formed in material weathered from metasedimentary rock (Natural Resources Conservation Service, 2017). Nine mid-sized diameter at breast height (60–75 cm DBH) Douglas-fir trees (average height 26 m, min: 20 m, max 35 m) were selected at random and spaced at least 500 m apart and within 30 m of unpaved access roads. Structurally unsound trees were avoided. Trees were climbed using minimally invasive and safe climbing techniques (Miller & Lowman, 2012; Anderson et al., 2015).

Where present, fist-sized (approx. 5 gram) or smaller patches of three epiphyte types (foliose lichens, fruticose lichens, and bryophyte) were collected and stored in paper bags (McCune et al., 1997). Sample collection was stratified into three sampling heights: below any branches on the bole (low, ∼10 m), the middle canopy (mid, ∼15 m), and the top 8 m of the tree (top, ∼25 m). Where possible one patch of foliose lichen and fruticose lichen were collected, and two bryophyte patches were collected from each tree at the three vertical locations. Not all epiphyte types were present at each sampling location and not all epiphytes were identified to species level.

### Processing samples for microfauna

A portion (0.3–1.9 grams, mean 0.95 grams) of each epiphyte sample was hydrated with 20 mL of commercially bottled spring water for 12 h to enable microfauna to become active. For each sample, three 1 mL aliquots were visually searched with a dissecting microscope at 20× magnification for nematodes, rotifers, and adult tardigrades. The abundance of nematodes, rotifers, and tardigrades was counted for each epiphyte sample and divided by the mass of the sample following Mitchell, Miller & Davis (2009) methodology.

Tardigrade specimens were deposited into a drop of polyvinyl alcohol media on a microslide (Salmon, 1951) with an Irwin loop (Schram & Davidson, 2012). A glass coverslip was placed over the medium, dried for three days, and nail polish was applied to seal the PVA mounting media.

### Species identification

Epiphytes were identified using standard techniques (Brodo, Sharnoff & Sharnoff, 2001) and identification guides (Norris & Shevock, 2004; McCune & Geiser, 2009) on a Zeiss 45–50–52 dissecting scope and a Leica ACT 2000 light microscope. Dominating genera for Bryophyte were: *Dicranum, Isothecium myosurides*; for foliose lichens: *Hypogymnia* and *Platismatia;* and for fruticose lichens: *Usnea*, *Sphaerophorus* (Table S1).

Tardigrades were identified using an Olympus BX60 DIC (differential interference contrast) microscope at 1,000× magnification. Morphological features including claws, buccopharyngeal apparatus, cuticle design, and other characteristics were used for species identification (Ramazzotti & Maucci, 1983; Pilato & Binda, 2010; Kaczmarek & Michalczyk, 2017; Stec et al., 2018). Nomenclature was based on Guidetti & Bertolani (2005), Degma & Guidetti (2007), Bertaloni et al. (2014) and Degma, Bertolani & Guidetti (2009–2018).

### Statistical analysis

Univariate three by three factorial analyses of variance for tardigrade, rotifer, and nematode density were blocked by tree with sampling height and epiphyte type as two interacting factors. The density of tardigrade, rotifer, or nematode populations and resulting microfaunal diversity per gram of epiphyte material was tested for differences across sampling heights, epiphyte types, and for interaction between sampling height and epiphyte type. Type III sum of squares was used for uneven replication and Tukey’s honestly significant difference was used to determine the magnitude and direction of statistical differences (*α* = 0.05). Pearson correlation coefficients of microfaunal density and mass of the portion of each sample did not reveal significant relationship of sample mass to microfaunal density (*p* > 0.98).

Tardigrade community composition was analyzed using a permutation multivariate analysis of variance (PERMANOVA) with the R package *vegan* (Oksanen et al., 2015). Dissimilarities in tardigrade community composition was visualized with non-metric multi-dimensionally scaled (NMDS) ordination using the Bray-Curtis dissimilarity method (Bray & Curtis, 1957; Dufrêne & Legendre, 1997). Ellipses representing 95% confidence intervals were displayed around the centroid of each epiphyte type to visualize significant differences in tardigrade composition. Tardigrade species richness was calculated as the count of each tardigrade taxa found in a sample. Tardigrade diversity and microfaunal diversity are reported using simpsons diversity index.

Additionally, each tardigrade species was tested for association with sampling heights or epiphyte types with the R package *indicspecies* (De Caceres & Legendre, 2009). All statistical analyses were performed with the program R ver. 3.3 (R Core Team, 2018), and visualized with the R package *ggplot2* (Wickham, 2009).

## Results

**Figure 2:**
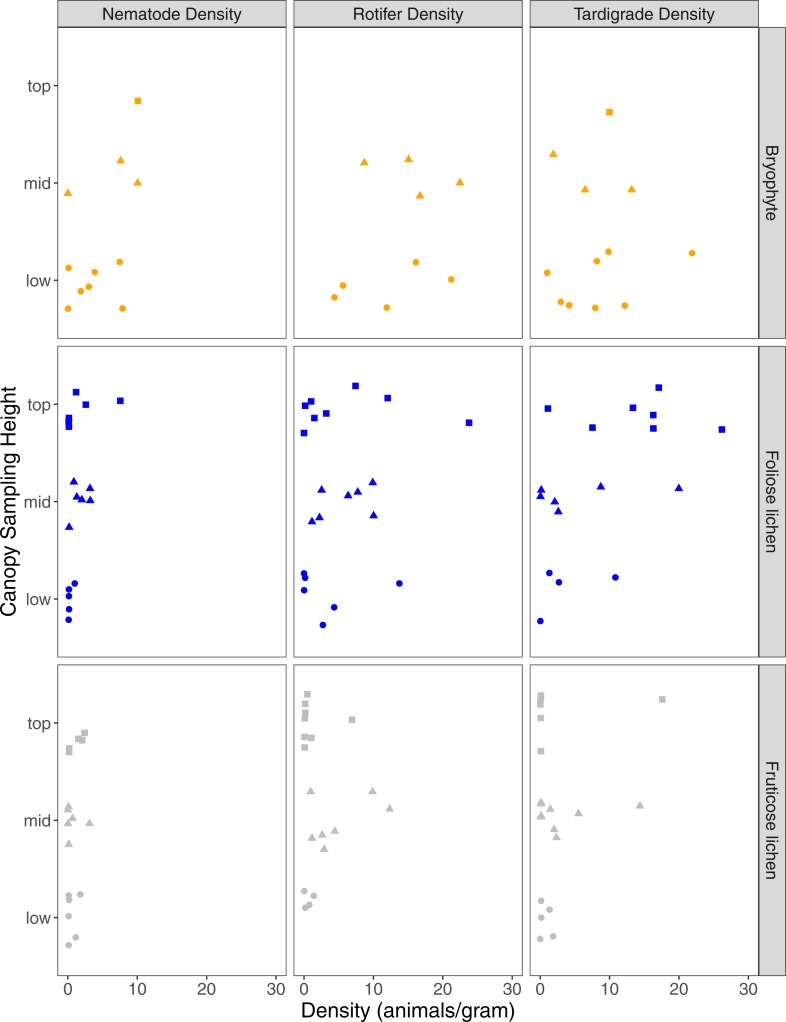
Nematode, rotifer, and tardigrade density (animals per gram) from three epiphyte types, and three canopy sampling locations within Douglas-fir trees. Square, triangle, circle data points represent microfauna density for samples that came from top, mid, or low sampling heights. Orange, blue or gray shapes represent bryophyte, foliose lichen, or fruticose lichen epiphyte types.

A total of 68 nematodes, 411 rotifers, and 231 tardigrades were found in 51 samples, with 89% of samples containing at least one nematode, rotifer, or tardigrade (Table 1). Analyzed epiphyte material included 18 lichen and bryophyte taxa (Table S1). Epiphyte type was significant in explaining differences in nematode (*p* = 0.03), rotifer (*p* = 0.01), and tardigrade density (*p* = 0.04). Nematode and rotifer density was higher in bryophytes than foliose lichen or fruticose lichen (*p* < 0.01), while tardigrade density was higher in bryophyte and foliose lichen than fruticose lichen (*p* < 0.01, *p* = 0.02). Microfaunal richness was also significantly higher in bryophytes and foliose lichen than fruticose lichen (*p* < 0.01). The mass of the portion of epiphyte used in analysis did not impact the density of nematodes, rotifers, or tardigrades (*p* = 0.98, *p* = 0.98, *p* = 0.99). Overall, nematodes were less common than rotifers or tardigrades (Fig. 2).

**Table 1:** Summary of microfauna density and epiphyte types. A summary of raw data that provides the % of positive samples, mean microfauna density, and tardigrade community data including species richness and average Simpson’s diversity index for each epiphyte type and sampling location.

Epiphyte type	Height	*N*	% positive samples			Density per sample			Tardigrade Community	
			Tardigrade	Rotifer	Nematode	Tardigrade	Rotifer	Nematode	Species	Diversity
						mean, sd	mean, sd	mean, sd	Richness	mean
Foliose lichen	Top	8	88%	88%	50%	15.4, 10.3	2.2, 2.6	0.6, 0.9	11	0.7
	Mid	9	89%	89%	67%	8.1, 6.5	1.3, 1.0	0.7, 0.7	7	0.5
	Low	7	29%	71%	14%	1.2, 1.1	2.7, 3.0	0.2, 0.4	7	0.3
Fruticose lichen	Top	8	50%	38%	38%	2.6, 6.1	0.4, 0.8	0.2, 0.3	3	0.3
	Mid	9	33%	56%	0%	0.9, 1.4	0.6, 1.0	0.0, 0.0	6	0.2
	Low	8	38%	25%	38%	1.0, 1.9	0.0, 0.2	0.1, 0.2	1	0.2
Bryophytes	Top	1	100%	100%	100%	14.3, NA	19.0, NA	3.3, NA	4	1.3
	Mid	4	75%	100%	50%	10.7, 10.9	5.3, 1.9	1.5, 1.7	5	0.4
	Low	9	100%	89%	67%	17.9, 12.2	11.2, 15.9	1.0, 1.2	4	0.3

Sampling height had a significant interaction with epiphyte type on nematode density, with mid and top bryophytes having significantly higher nematode density than mid and top fruticose lichen (*p* = 0.03, *p* < 0.01). However, rotifer and tardigrade density were not significantly impacted by sampling height (*p* = 0.6, *p* = 0.63). Of the nine trees accessed, only one bryophyte was found in the top sampling location which also supported the highest nematode density in the collection (Fig. 2). Three tardigrade species were significantly associated with the top sampling height (*Pilatobius nodulosus, p* > 0.01; *Echiniscus quadrispinosus*, *p* = 0.03; *Milnesium sp. 2, p* = 0.04), and *Ramazzottius oberhauseri* significantly associated with the top and mid sampling heights (*p* = 0.05). Epiphyte species richness was lowest in the top position (6 species), and similar at the middle (11 species) and lowest sampling height (10 species).

**Figure 3:**
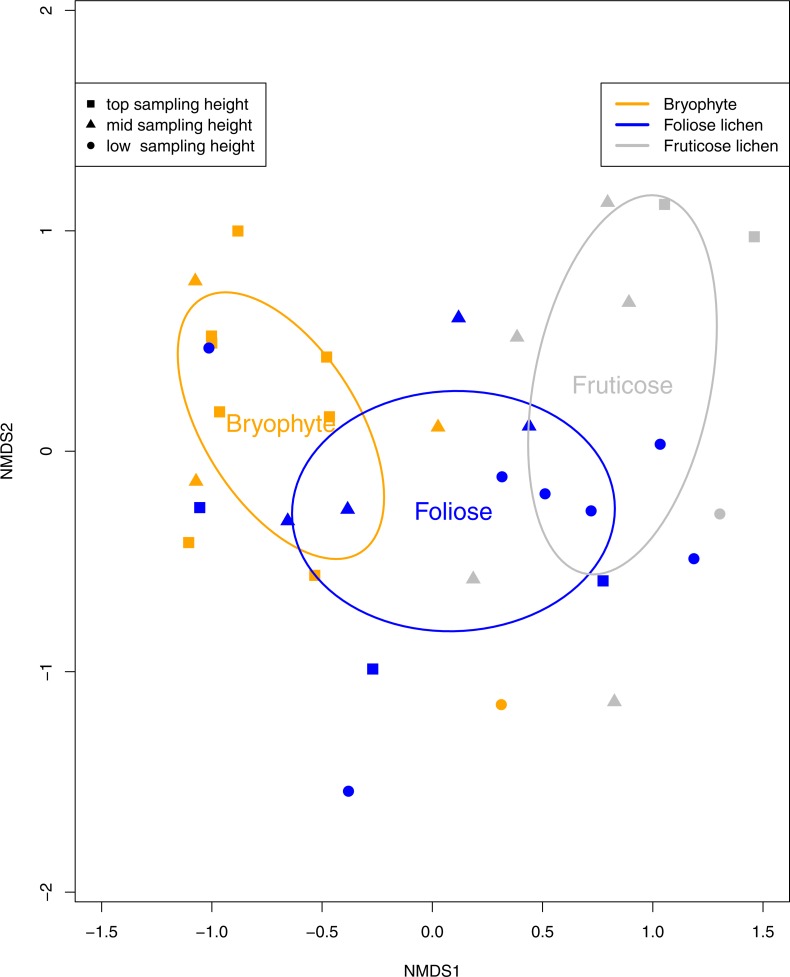
A non-metric multi-dimensional scaling (NMDS) ordination of tardigrade community composition with 95% confidence ellipses surrounding Bryophyte, Foliose lichen, and Fruticose lichen samples. Each data point represents a bryophyte (orange), foliose lichen (blue), or fruticose lichen (gray) sample. Squares represent samples from the top sampling location, triangles represent the mid sampling location, and circles represent the low sampling location. Similarity between the tardigrade communities found in each sample can be interpreted by the proximity of each symbol. Tardigrade communities were significantly different between epiphyte types (*p* < 0.01) and sampling heights (*p* = 0.02).

Eight species of tardigrade and four tardigrade taxa were found, representing two classes, three orders, six families and eight genera (Table 2). Tardigrade taxa in the *Macrobiotus hufelandi* group comprised 31% of the collection, while the second most common species, *Echiniscus quadrispinosus* compromised 22% of the collection. Tardigrade diversity was significantly higher in fruticose lichen than bryophyte and foliose samples (*p* < 0.01). Furthermore, tardigrade community composition was significantly impacted by epiphyte type (*p* < 0.01, Fig. 3) and sampling height (*p* = 0.02). The *Macrobiotus hufelandi* group was significantly associated with bryophyte samples (*p* < 0.01). In contrast, tardigrade species *Echiniscus quadrispinosus* and *Ramazzottius oberhauseri* were significantly associated with foliose lichen, and fruticose lichen (*p* < 0.01, *p* = 0.02).

**Table 2:** Tardigrade species found.

Class, Order, SuperFamily, Family	Foliose Lichen	Bryophytes	Fruticose Lichen	Total
Genus species	*n* = 22	*n* = 14	*n* = 27	*N* = 63
	mean, sd	mean, sd	mean, sd	
Eutardigrada, Apochela, Milnesiidae				
*Milnesium eurystomum* (Maucci, 1991)	0.1, 0.2	0	0.8, 1.5	4
*Milnesium* sp.1	0.1, 0.3	0.1, 0.3	0.3, 0.5	9
*Milnesium* sp. 2	0.3, 0.8	0.3, 0.9	0	4
Eutardigrada, Parachela, Hypsibiidae				
*Pilatobius nodulosus* (Ramazzotti, 1957)	0.6, 1.2	0.3, 0.6	0	14
Eutardigrada, Parachela, Itaquasconinae				
*Itaquascon* sp.	0.1, 0.2	0	0	1
Eutardigrada, Parachela, Isohypsibidea, Isohypsibioiidae				
*Ramazzottius sp.* (Doyere, 1840)	1.0, 1.4	0.1, 0.3	1.0, 1.4	24
Eutardigrada, Parachela, Macrobiotoidea, Macrobiotidae				
*Macrobiotus hufelandii* group	0.7, 1.4	4.9, 6.1	0	70
*Mesobiotus harmsworthi* (Murray, 1907)	0.9, 1.7	0.5, 0.9	0	22
Heterotardigrada, Echiniscoidea, Echiniscoididae				
*Echiniscus arctomys group* (Ehrenberg, 1853)	0.6, 1.0	0.2, 0.4	0	12
*Echiniscus horningi* ( Schuster & Grigarick, 1971)	0.8, 2.0	0	0	15
*Echiniscus quadrispinosus* (Richters, 1902)	2.4, 3.1	0.1, 0.3	1.3, 1.3	50
*Multipseudechinisus raneyi* (Grigarick, Mihelčič, & Schuster 1964)	0.2, 0.6	0.1, 0.3	0	15

**Notes.**

Mean average density of each species in each epiphyte type sd standard deviation *n* the number of epiphyte type samples *N* total samples

All identifications are based on morphological approaches.

## Discussion

Tardigrades, nematodes, and rotifers had differential distributions across the three sampling heights and epiphyte types studied, suggesting varying habitat suitability for different phyla. These patterns may be due to epiphyte water-retention characteristics, secreted secondary metabolites, light availability (Wright, 1991), or food availability and feeding habit limitations (e.g., filter feeding rotifers require higher humidity to feed) (Guidetti et al., 2012; Hallas & Yeates, 1972). While microfaunal populations varied substantially within and between trees a general trend was that fewer nematodes, rotifers, and tardigrades were found in the fruticose lichen. In contrast, overall microfaunal density was highest in bryophytes growing in the low sampling height, suggesting that the consistently humid habitat combined with optimal resource availability (e.g., photosynthetic cells) is sufficient for microfaunal communities to thrive and epiphyte growth form is relevant to microfaunal communities (Jönsson, 2003).

Nematode, rotifer, and tardigrade density had contrasting responses to sampling height, with nematode density increasing with sample height, while rotifer and tardigrade density was not significantly different across sampling heights. The trend we report has not been found for Nematoda, but previous studies document similar responses of tardigrade density to tree height (Miller, Gallardo & Clark, 2013; Chang et al., 2015) and four tardigrade species significantly associated with top canopy positions suggests that microfaunal communities are likely impacted by sampling height, in addition to epiphyte type.

Although fruticose lichens were sparsely populated, *Echiniscus quadrispinosus* and *Ramazzottius oberhaeuseri* were mainly found in fruticose lichens over bryophyte and foliose lichen (Table 1). Two of the four tardigrade species found in fruticose samples feed on microbes (*Echiniscus quadrispinosus* and *Ramazzottius oberhaeuseri*) while the other two are predatory (*Milnesium eurystomum* and *Milnesium.* sp 1)*.* The “hair-lichen” morphology of fruticose lichen may represent a relatively xeric, high-stress environment, with implications for those taxa which can successfully colonize (Grime, 1977; Guil et al., 2009; Bartels, Nelson & Exline, 2011). All of the tardigrade taxa found in this study were documented in Schuster & Grigarick (1965) and  Schuster & Grigarick (1971) suggesting population stability during the 50 years between each study. This highlights the utility of co-locating field sites with previous studies to inform rates of long-term immigration and emigration and provides further evidence that phylum Tardigrada is relatively species poor (Bartels et al., 2016).

Epiphytes in tree canopies seem to support a similar tardigrade species richness as epiphytes found at ground level, and our observation of higher microfaunal diversity in bryophytes than lichen is supported by Bartels & Nelson (2007), Guil et al. (2009), Zawierucha et al. (2016) and Zawierucha et al. (2017). An understanding of regional tardigrade community structure in North America is beginning to emerge (Meyer, 2013; Kaczmarek, Michalczyk & McInnes, 2016).

The distribution of microfaunal communities is complex, but may be explained by immigration events (Mogle et al., 2018; Zawierucha et al., 2018), reproduction rates (Tsujimoto, Imura & Kanda, 2016; Bingemer, Hohberg & Schill, 2016), lifespan and life history traits (Schuster & Greven, 2013), suitable abiotic environment (Wright, 1989), and site specific biotic factors (Kinchin, 1994; Guil et al., 2009; Glime, 2013) including feeding behavior (Guil et al., 2009; Sánchez-Moreno, Ferris & Guil, 2008; Miller, Horning & Heatwole, 2001; Guidetti et al., 2012; Guil & Sanchez-Moreno, 2013). Although there are likely numerous interacting forces that contribute to microfaunal species distributions, disentangling the impact of epiphyte growth form and abiotic micro-climate on microfaunal abundance is challenging because epiphyte species and microfaunal distributions may respond similarly to gradients. Experimental manipulations of habitat characteristics using factorial treatment designs may be useful to decipher microfaunal habitat preferences. Habitat manipulations could help identify mechanisms driving microfaunal distribution.

## Conclusion

Microfaunal populations respond to epiphyte type, and to a lesser extent, sampling height. This evidence suggests that microfauna are more strongly influenced by biotic micro-environmental forces such as epiphyte growth form and water retention characteristics than abiotic micro-environmental forces alone. Additionally, it may be more informative to view microfaunal population dynamics through the lens of their habitat morphology, as conserved functional traits of epiphyte morphology may mirror local micro-environmental forces on which microfaunal micro-population establishment is dependent. Future ecological studies on microfauna could benefit from carefully considering epiphyte morphology.

##  Supplemental Information

10.7717/peerj.5699/supp-1 Supplemental Information 1 R code for all analyses and graphics for “Microfauna response to epiphyte type and sampling height” This reproduce-able R code is meant to show all analyses and graphics included in the corresponding manuscript. There are seven components. # # #1. Read in dataset, calculate Simpsons diversity index. # # #2. Results for Microfauna density ANOVA # # #3. Test for correlation of mass and microfauna density # # #4. Figure 2. Scatter plots of microfauna Density. # # #5. Figure 3. NMDS on tardigrade community # # #6. Result for permanova (adonis) of tardigrade community composition # # #7. Indicator species analysis

10.7717/peerj.5699/supp-2 Table S1 Epiphyte species stratified at three sampling heights This list details the epiphyte species found in the canopy of nine Douglas-fir trees in Six River’s National Park across three sampling heights.

10.7717/peerj.5699/supp-3 Supplemental Information 2 Raw data that all analyses were based on

## Additional information and Declarations

### Data Availability

The following information was supplied regarding data availability: The raw data are provided in the Supplemental Files.
